# Association between intensive care unit admission of a patient and mental disorders in the spouse: a retrospective matched-pair cohort study

**DOI:** 10.1186/s40560-021-00583-3

**Published:** 2021-10-29

**Authors:** Yuki Miyamoto, Hiroyuki Ohbe, Tadahiro Goto, Hideo Yasunaga

**Affiliations:** 1 Department of Clinical Epidemiology and Health Economics, School of Public Health, The University of Tokyo, 7-3-1 Hongo, Bunkyo-ku, Tokyo, 1130033 Japan; 2 Department of Emergency Medicine, Kyoto Prefectural University of Medicine, Kaji-cho 465, Kamigyo-ku, Kyoto, 6028566 Japan; 3 TXP Medical Co. Ltd., 7-3-1-252 Hongo, Bunkyo-ku, Tokyo, 1138454 Japan

**Keywords:** Mental disorders, Post-intensive care syndrome, Administrative claim database, Post-intensive care syndrome-family, PICS-F

## Abstract

**Background:**

Previous prospective studies have suggested that spouses of patients who are admitted to the intensive care unit (ICU) have a high prevalence of mental disorders, termed post-intensive care syndrome-family (PICS-F). However, it remains unclear whether the patient’s ICU admission is associated with the occurrence of mental disorders in the spouse outside of the prospective study setting. We therefore investigated the proportion of ICU patients’ spouses who visited medical facilities for mental disorders and the association between ICU admission of a patient and mental disorders in the spouse using real-world data.

**Methods:**

This was a retrospective matched-pair cohort study using commercially available, routinely collected administrative claims data. As the study population, we identified all married couples (both wife and husband) who were registered in the database from 1 April 2012 to 31 August 2018 using family identification codes. We identified spouses of patients who were admitted to the ICU for more than 2 days as the exposure group and defined the date of admission to the ICU as the index date. We randomly matched four individuals in the non-exposure group with one individual in the exposure group. The primary outcome was any PICS-F–related mental disorder in the spouses within 6 months from the index date. As a sensitivity analysis, we also investigated the proportion and association of individuals (excluding spouses) with a history of mental disorders.

**Results:**

Among 1,082,208 married couples, we identified 8490 spouses of ICU patients, and they were matched with 33,946 individuals. The proportion of any PICS-F–related mental disorder within 6 months from the index date was 12.8% in ICU patients’ spouses and 11.3% in the matched individuals (adjusted odds ratio, 1.29; 95% confidence interval, 1.03–1.42). The sensitivity analysis showed significant associations between ICU admission and spouses’ mental disorders.

**Conclusions:**

Spouses of patients who were admitted to the ICU had a slightly higher risk of mental disorders within 6 months than spouses of patients who were not admitted to the ICU.

**Supplementary Information:**

The online version contains supplementary material available at 10.1186/s40560-021-00583-3.

## Background

Family members of patients in intensive care units (ICUs) can be physically and psychologically affected because of the patients’ unexpected situation and uncertain clinical outcomes [1, 2]. The resultant stress experienced by such patients’ family members can lead to sleep disorders, anxiety, depression, and post-traumatic disorder, some of which may persist for months after the patient’s ICU discharge [1–3]. These problems are termed post-intensive care syndrome-family (PICS-F) [4–6], and spouses may be the most susceptible to PICS-F among all family members [7].

Many studies have shown that family members of patients in the ICU have a high prevalence of mental disorders [7–16]. All of these studies were prospectively designed and based on self-reported questionnaires or structured interviews (e.g., Hospital Anxiety and Depression Scale). The reported prevalence of anxiety disorders, depression, and post-traumatic stress disorders in these studies ranged from 10 to 67%, 16 to 56%, and 14 to 69%, respectively [7–16]. Additionally, one study showed that about 40% of family members of patients in the ICU experienced at least one mental disorder [7].

Whether this high prevalence of mental disorders among family members was actually attributed to the patient’s ICU admission remains unclear because these studies did not have a control population. Moreover, whether family members of patients in the ICU have mental disorders outside research settings is unknown because the psychological status of family members in this situation is not routinely assessed in the real-world clinical setting. Several interventions for family members of critically ill patients (e.g., family conference, flexible family presence policy, and brochures for families) were recently shown to be effective; however, none of them are routinely performed or covered by health insurance [4, 17, 18].

Therefore, in this matched cohort study using a real-world database, we evaluated the proportion of ICU patients’ spouses who visited medical facilities for mental disorders and the association between ICU admission of a patient and mental disorders in the patient’s spouse.

## Methods

This retrospective matched-pair cohort study was performed using commercially available, routinely collected administrative claims data. The Institutional Review Board of The University of Tokyo approved the study protocol (10862-[1]). The anonymous nature of the data allowed the requirement for informed consent from the patients to be waived.

### Data source

The data were obtained from the Japan Medical Data Center (JMDC Inc., Tokyo, Japan), which has collected data from more than 60 health insurers since 2005. The database includes both outpatient and inpatient health insurance claims data on more than 6,000,000 individuals. The JMDC provides researchers these claims data after de-identification. Most individuals are employees and their dependents of relatively large Japanese companies. Because Japanese people aged > 75 years shift their insurance to national health insurance for the elderly (called the Medical Care System for the Latter-Stage Elderly), almost all individuals registered to the database are non-elderly or young [19].

The database contains the following information: (1) patient characteristics including age, sex, birthdate, date of insurance registration, and date of insurance withdrawal; (2) status of medical insurance (insured or dependent with insured person), family identification codes, and relationship with insured person (e.g., spouse, child, or parent); (3) dates of outpatient clinic or hospital visits; and (4) codes and dates of diagnoses, prescribed drugs, and medical procedures. Diagnoses are recorded based on the International Classification of Diseases, Tenth Revision (ICD-10); drugs are recorded based on the Anatomical Therapeutic Chemical Classification System (ATC); and procedures are recorded based on Japanese medical procedure codes. Because the dates of prescriptions have been recorded since 1 April 2012, we used data from 1 April 2012 to 31 August 2018.

### Study population and matched cohort

As the study population, we identified all married couples (both wife and husband) who were registered in the database from 1 April 2012 to 31 August 2018 using family identification codes. Among the study population, we included patients who were admitted to the ICU for the first time and stayed for at least 2 consecutive days; they were defined as “ICU patients.” We set the date of the first admission to the ICU as the index date. The exposure group was defined as the spouses of the patients who were admitted to the ICU; they were defined as “spouses of ICU patients” or “ICU patients’ spouses.” We excluded the following spouses of ICU patients from the exposure group: (1) those who were admitted to the ICU on or before the index date, (2) those who were registered in the database after the index date, and (3) those who withdrew their health insurance before the index date. In the same manner, we included the following individuals in the non-exposure group: (1) those who were not admitted to the ICU on or before the index date and (2) those whose spouses were not admitted to the ICU on or before the index date. Among these study populations, spouses of ICU patients were randomly matched to individuals in the non-exposure group with an exposure to non-exposure ratio of 1:4; matching was performed according to age (the same month and year of birth), sex, and status of medical insurance; they were defined as “matched individuals.” Their spouses were termed “spouses of matched individuals.” Using this method, individuals in the exposure group before the index date could be selected as individuals in the non-exposure group, and the individuals could be selected as the non-exposure group several times.

### Outcomes and variables

The primary outcome was the proportion of ICU patients’ spouses and matched individuals who visited medical facilities for any mental disorders related to PICS-F at least once within 6 months after the index date of the matched pair. In this study, mental disorders related to PICS-F were defined as diagnoses with ICD-10 codes of anxiety disorders (F40–F42), mood disorders (F30–F39), post-traumatic stress disorders (F43), and sleep disorders (F51, G47) [20]. The secondary outcomes were the proportion of the spouses who received anxiolytics, hypnotics/sedatives (ATC codes N05B/N05C), or antidepressants (ATC code N06A). We also evaluated primary and secondary outcomes from the index date to 1 month, 1 to 2 months, 2 to 3 months, 3 to 4 months, 4 to 5 months, and 5 to 6 months.

Covariates included the following variables of ICU patients’ spouses and matched individuals: age, sex, status of medical insurance, history of sleep disorders, history of anxiety disorders, history of mood disorders, history of post-traumatic stress disorders, and Charlson comorbidity index score [10, 21]. The definitions of these histories of mental disorders and the Charlson comorbidity index were based on ICD-10 codes (Additional file 1: Table S1) when they visited at least once during the 6 months before the index date [22]. We also evaluated the characteristics of the ICU patients. The definitions of the main diagnoses necessitating ICU admission were based on ICD-10 codes (see Additional file 1: Table S2).

### Statistical analysis

Categorical variables are presented as number and percentage, and continuous variables are presented as mean and standard deviation (SD) or median and interquartile range (IQR) as appropriate. The baseline characteristics of matched pairs with and without exposure were compared using the *χ*^2^ test for binary variables and the *t*-test for normally distributed continuous variables or the Wilcoxon rank-sum test for skewed continuous variables. In the matched-pair cohort, we performed multivariable conditional logistic regression analyses on the primary and secondary outcomes for each interval to estimate the odds ratios (ORs) and 95% confidence intervals (CIs), with adjustment for age, sex, status of medical insurance, history of anxiety disorders, history of mood disorders, history of sleep disorders, and Charlson comorbidity index score. We also graphically described the monthly proportions of secondary outcomes during the 6 months before and after the index date.

Additionally, we performed three sensitivity analyses of the primary and secondary outcomes. First, to assess the effect of ICU admission on new-onset mental disorders in spouses of ICU patients and matched individuals, we excluded those with a history of mental disorders. Second, we excluded spouses of ICU patients and matched individuals whose spouses (i.e., ICU patients and spouses of matched individuals) died within 6 months after the index date because many studies have shown that bereavement can cause mental disorders in spouses [23–26]. In other words, bereavement was considered to be a potential mediator of spouses’ mental disorders in our study. Third, to reduce bias caused by withdrawal of insurance due to serious illness, financial burdens, and psychological problems, we excluded spouses of ICU patients and matched individuals who withdrew their insurance within 6 months after the index date.

All P-values were two-tailed, and a *P* value of < 0.05 indicated a statistically significant difference. Analyses were performed using Stata/MP version 16 (StataCorp, College Station, TX, USA) and R statistical software version 3.6.2 (The R Foundation for Statistical Computing, Vienna, Austria).

## Results

### Patient selection and baseline characteristics

**Fig. 1:**
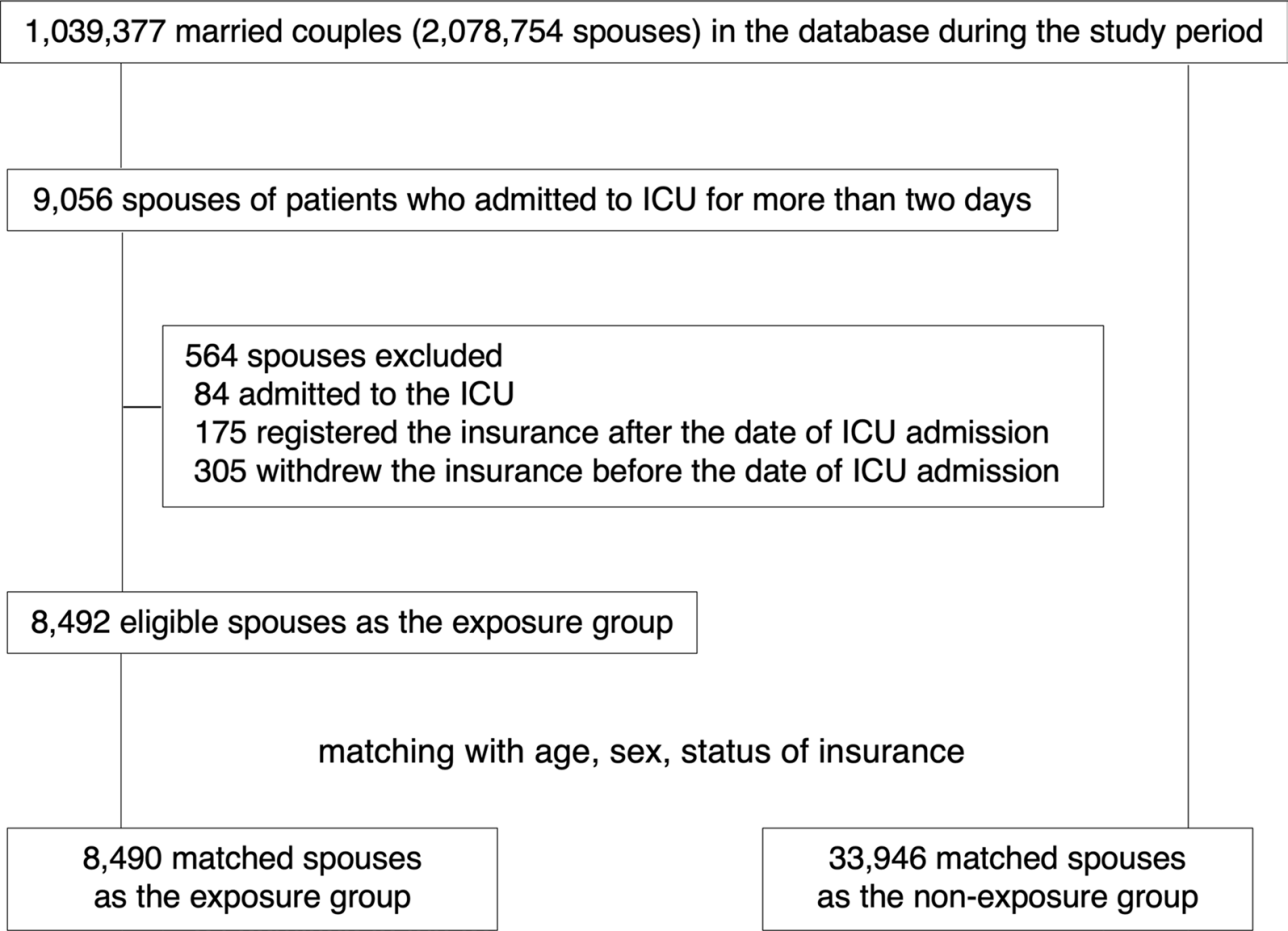
Patient selection

Among 1,039,377 married couples (2,078,754 spouses) in the database during the study period, we identified 9056 (0.4%) spouses of patients who were admitted to ICU for more than 2 days (Fig. 1). After excluding 564 patients, we identified 8492 eligible individuals as the exposure group. Of these, 8490 spouses of ICU patients were matched with 33,946 individuals.

The baseline characteristics of the 8,490 spouses of ICU patients and the 33,946 matched individuals are shown in Table 1. Their mean age was 53.8 (SD, 9.9) years, the proportion of male individuals was 35.3%, and the proportion of individuals who were dependent on their spouse’s medical insurance was 63.4% in both groups. There were significant differences in spouses’ history of sleep disorders and mood disorders between the two groups. The mean observational period in the two groups was 59.5 and 63.1 months, respectively. The proportion of spouses who withdrew their insurance within 6 months after the index date was 20.9% in the ICU patients’ spouses group, which was significantly higher than that in the matched individuals group.

**Table 1:** Baseline characteristics of ICU patients’ spouses and matched individuals

Characteristics	ICU patients’ spouses (*n* = 8490)	Matched individuals (*n* = 33,946)	*P* value
Age, years, mean (SD)	53.8 (9.9)	53.8 (9.9)	0.98
Male	2996 (35.3)	11,981 (35.3)	0.99
Medical insurance			0.98
Independents	3108 (36.7)	12,421 (36.6)	
Dependents	5382 (63.4)	21,525 (63.4)	
History of sleep disorders	827 (9.7)	2883 (8.5)	< 0.001
History of mood disorders	370 (4.4)	1275 (3.8)	0.01
History of anxiety disorders	279 (3.3)	1039 (3.1)	0.28
Charlson comorbidity index			0.46
0	5336 (62.9)	21,599 (63.6)	
1	1250 (14.7)	5028 (14.8)	
2	860 (10.1)	3341 (9.8)	
3	468 (5.5)	1829 (5.4)	
≥ 4	576 (6.8)	2149 (6.3)	
Withdrew insurance within 6 months	1774 (20.9)	5183 (15.3)	< 0.001

Unless otherwise stated, data are presented as number (%)

*ICU* intensive care unit; *SD* standard deviation

The baseline characteristics of the 8,490 ICU patients are shown in Additional file 1: Table S3. Their mean age was 54.9 (SD, 9.9) years, and 35.3% were female. The leading cause of ICU admission was sepsis (53.2%). The proportion of patients who received mechanical ventilation was 30.8%. The proportion of ICU patients who died within 6 months after the index date was 6.9%. The median length of stay was 16 days (IQR 10–28 days), and the median length of ICU stay was 3 days (IQR 2–7 days).

### Primary and secondary outcomes

Table 2 shows the proportion of ICU patients’ spouses and the proportion of matched individuals who visited medical facilities for mental disorders related to PICS-F as well as the adjusted ORs and their 95% CIs for each time interval. The proportion of ICU patients’ spouses within 6 months from the index date was 12.8%, and that of matched individuals was 11.3% (adjusted OR 1.29; 95% CI 1.03–1.42). There was no significant difference in the proportions in each time period between the two groups.

**Table 2:** Proportions of ICU patients’ spouses and matched individuals who visited medical facilities for any mental disorders related to post-intensive care syndrome-family

Period of time	ICU patients’ spouses (*n* = 8490)	Matched individuals (*n* = 33,946)	Adjusted odds ratio (95% CI)*	*P* value
Overall (index date to 6 months)	1084 (12.8)	3819 (11.3)	1.29 (1.03–1.42)	0.02
Index date to 1 month	706 (8.3)	2462 (7.3)	1.15 (0.92–1.43)	0.20
1–2 months	650 (7.7)	2434 (7.2)	1.17 (0.97–1.42)	0.10
2–3 months	604 (7.1)	2337 (6.9)	0.97 (0.81–1.17)	0.77
3–4 months	614 (7.2)	2321 (6.8)	1.03 (0.86–1.23)	0.73
4–5 months	581 (6.8)	2211 (6.5)	0.93 (0.78–1.11)	0.44
5–6 months	562 (6.6)	2190 (6.5)	0.92 (0.78–1.10)	0.38

Data are presented as *n* (%)

*ICU* intensive care unit; *CI* confidence interval

*Adjusted for age, sex, status of medical insurance, history of sleep disorders, history of anxiety disorders, history of mood disorders, history of post-traumatic stress disorders, and Charlson comorbidity index score

**Fig. 2:**
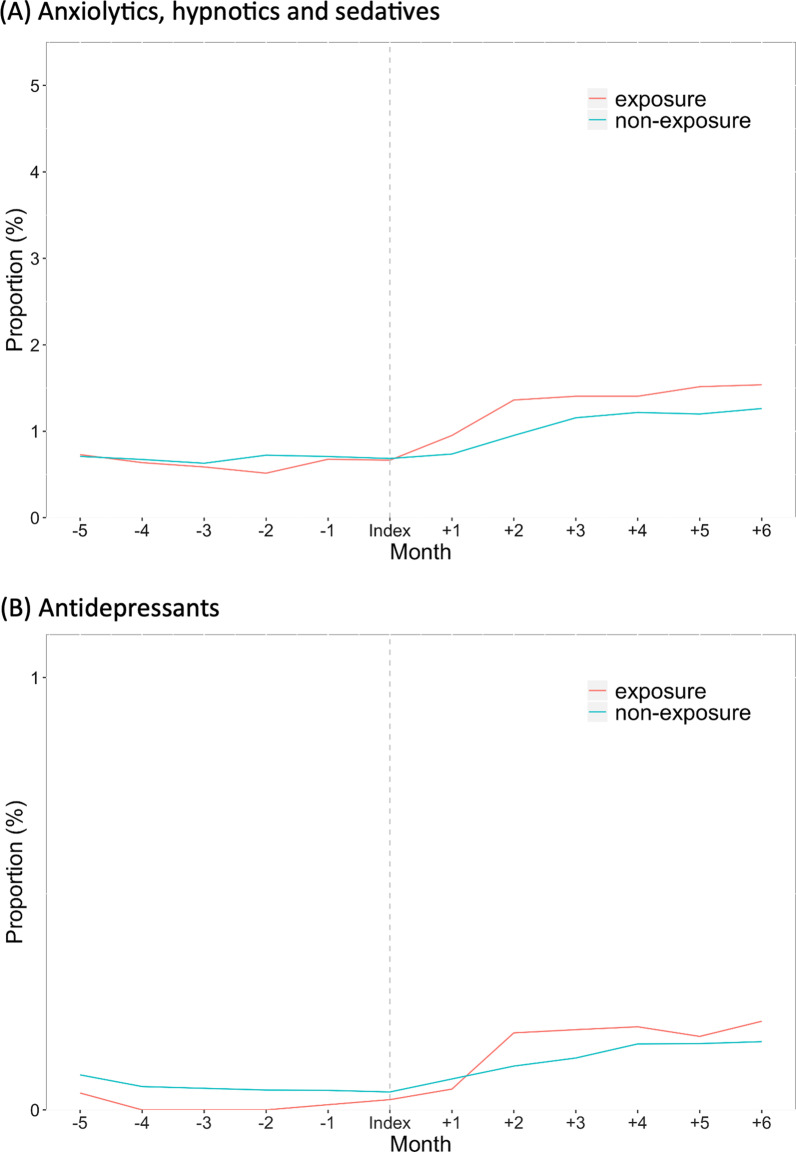
Proportion of ICU patients’ spouses and matched individuals who were prescribed psychotropic medications in the 6 months before and after the index date. Monthly percentages are based on a denominator of patients still registered in that month

The overall and monthly proportions of spouses who received anxiolytics, hypnotics/sedatives, and antidepressants are shown in Table 3. The proportion of ICU patients’ spouses who received anxiolytics and hypnotics/sedatives within 6 months from the index date was 12.7%, and that of matched individuals was 10.7% (adjusted OR 1.19; 95% CI 1.07–1.32). The crude proportion of ICU patients’ spouses who received anxiolytics and hypnotics/sedatives increased during the first 2 months and then decreased; the crude proportion thereafter remained higher than that of matched individuals before the index date after ICU admission (Fig. 2A). The proportions were significantly higher from 0 to 1 month (8.5% vs. 6.7%; adjusted OR 1.32; 95% CI 1.13–1.54) and from 1 to 2 months (8.0% vs. 6.6%; adjusted OR 1.29; 95% CI 1.12–1.49) from the index date, whereas there were no significant differences from 2 to 3 months, from 3 to 4 months, from 4 to 5 months, or from 5 to 6 months (Table 3).

**Table 3:** Proportions of ICU patients’ spouses and matched individuals who were prescribed anxiolytics, hypnotics/sedatives, or antidepressants

Period of time	ICU patients’ spouses (*n* = 8490)	Matched individuals (*n* = 33,946)	Adjusted odds ratio (95% CI) *	*P* value
*Anxiolytics and hypnotics/sedatives*				
Overall (prescribed within 6 months)	1081 (12.7)	3619 (10.7)	1.19 (1.07–1.32)	0.001
Index date to 1 month	724 (8.5)	2288 (6.7)	1.32 (1.13–1.54)	< 0.001
1–2 months	680 (8.0)	2226 (6.6)	1.29 (1.12–1.49)	0.001
2–3 months	602 (7.1)	2218 (6.5)	1.03 (0.90–1.19)	0.645
3–4 months	614 (7.2)	2132 (6.3)	1.01 (0.88–1.17)	0.85
4–5 months	565 (6.7)	2211 (6.0)	1.08 (0.93–1.25)	0.32
5–6 months	548 (6.5)	2024 (6.0)	1.02 (0.88–1.18)	0.78
*Antidepressants*				
Overall (prescribed within 6 months)	233 (2.7)	838 (2.5)	1.15 (0.85–1.56)	0.37
Index date to 1 month	190 (2.2)	700 (2.1)	0.96 (0.61–1.52)	0.86
1–2 months	181 (2.1)	682 (2.0)	1.22 (0.82–1.83)	0.31
2–3 months	167 (2.0)	666 (2.0)	1.14 (0.78–1.68)	0.50
3–4 months	153 (1.8)	650 (1.9)	0.90 (0.61–1.33)	0.60
4–5 months	148 (1.7)	620 (1.8)	0.95 (0.64–1.40)	0.79
5–6 months	143 (1.7)	606 (1.8)	0.97 (0.65–1.43)	0.87

Data are presented as *n* (%)

*ICU* intensive care unit; *CI* confidence interval

*Adjusted for age, sex, status of medical insurance, history of sleep disorders, history of anxiety disorders, history of mood disorders, history of post-traumatic stress disorders, and Charlson comorbidity index score

The proportion of ICU patients’ spouses who received antidepressants within 6 months from the index date was 2.7%, and that of matched individuals was 2.5% (adjusted OR 1.15; 95% CI 0.85–1.56). The crude proportions of ICU patients’ spouses who received antidepressants were similar before and after the index date between the two groups (Fig. 2B). There were no statistically significant differences in the proportions within each monthly interval between the two groups (Table 3).

### Sensitivity analyses

**Fig. 3:**
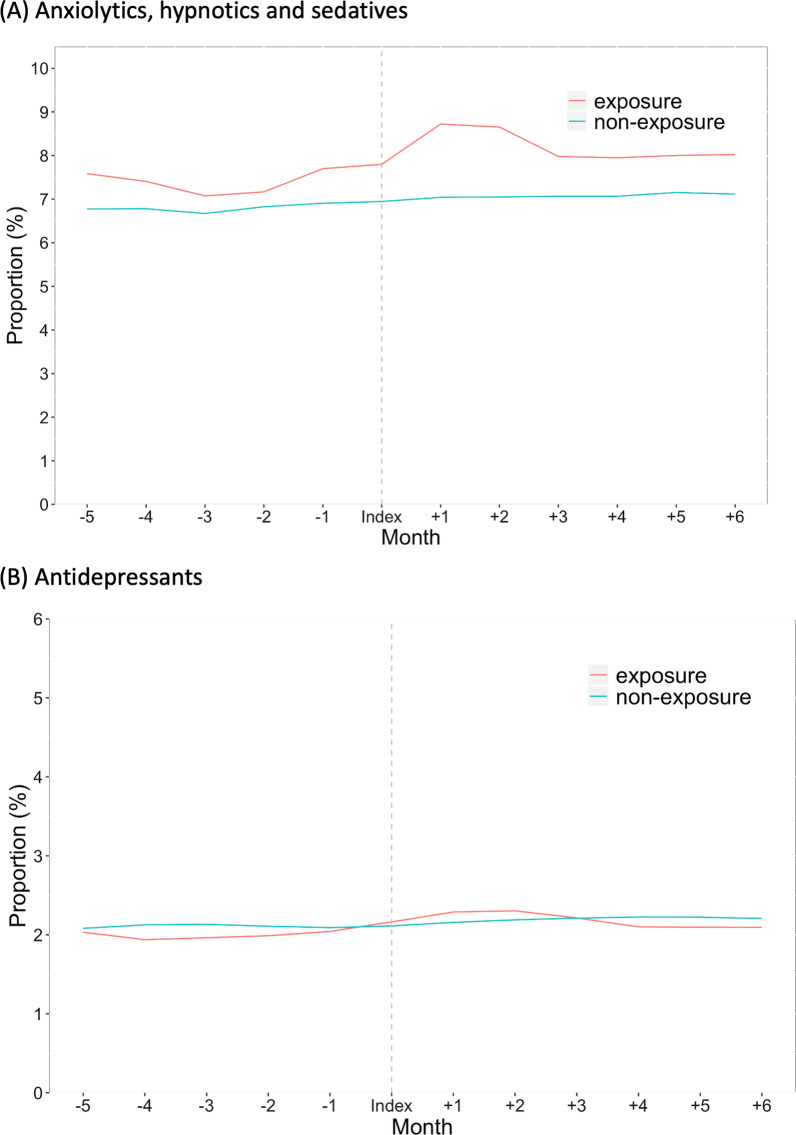
Proportion of ICU patients’ spouses and matched individuals who did not have a history of mental disorders and who were prescribed psychotropic medications in the 6 months before and after the index date. Monthly percentages are based on a denominator of patients still registered in that month

In the sensitivity analysis that excluded individuals with a history of mental disorders, 1242 and 4523 individuals with a history of any mental disorder were excluded from spouses of ICU patients and matched individuals, respectively. The proportions of spouses newly diagnosed with any mental disorders related to PICS-F in each month were significantly higher in the exposure group than in the non-exposure group both overall and within each monthly interval (Table 4). Regarding the secondary outcomes in this sensitivity analysis, the crude proportion of ICU patients’ spouses and matched individuals who received anxiolytics and hypnotics/sedatives showed a similar change (Fig. 3A). In the multivariable logistic regression analyses, the proportion of ICU patients’ spouses who received anxiolytics and hypnotics/sedatives was significantly higher than that of matched individuals until 2 months from the index date. However, there were no significant differences after 2 months from the index date (Table 5). The crude proportion of ICU patients’ spouses who received antidepressants was slightly higher than that of matched individuals; however, in the multivariable logistic regression analyses, there were no significant differences in the proportions between the two groups (Fig. 3B, Table 5).

**Table 4:** Proportions of ICU patients’ spouses and matched individuals without a history of mental disorders who visited medical facilities for any mental disorders related to post-intensive care syndrome-family

Period of time	ICU patients’ spouses (*n* = 7248)	Matched individuals (*n* = 29,423)	Adjusted odds ratio (95% CI)*	*P* value
Overall (index date to 6 months)	162 (2.2)	471 (1.6)	1.41 (1.17–1.70)	< 0.001
Index date to 1 month	24 (0.3)	50 (0.2)	2.07 (1.23–3.47)	0.006
1–2 months	56 (0.8)	102 (0.3)	2.20 (1.57–3.10)	< 0.001
2–3 months	55 (0.8)	142 (0.5)	1.56 (1.12–2.15)	0.007
3–4 months	75 (1.0)	178 (0.6)	1.75 (1.32–2.32)	< 0.001
4–5 months	69 (1.0)	182 (0.6)	1.61 (1.20–2.14)	0.001
5–6 months	68 (0.9)	211 (0.7)	1.34 (1.01–1.80)	0.04

Data are presented as *n* (%)

*ICU* intensive care unit; *CI* confidence interval

*Adjusted for age, sex, status of medical insurance, history of sleep disorders, history of anxiety disorders, history of mood disorders, history of post-traumatic stress disorders, and Charlson comorbidity index score

**Table 5:** Proportions of ICU patients’ spouses and matched individuals without a history of mental disorders who were prescribed anxiolytics, hypnotics/sedatives, or antidepressants

Period of time	ICU patients’ spouses (*n* = 7248)	Matched individuals (*n* = 29,423)	Adjusted odds ratio (95% CI) *	*P* value
*Anxiolytics and hypnotics/sedatives*				
Overall (prescribed within 6 months)	323 (4.5)	1,143 (3.9)	1.16 (1.01–1.32)	0.03
Index date to 1 month	79 (1.1)	247 (0.8)	1.33 (1.01–1.75)	0.04
1–2 months	107 (1.5)	310 (1.1)	1.40 (1.11–1.77)	0.004
2–3 months	106 (1.5)	366 (1.2)	1.15 (0.92–1.45)	0.20
3–4 months	102 (1.4)	375 (1.3)	1.13 (0.90–1.41)	0.30
4–5 months	107 (1.5)	360 (1.2)	1.25 (1.00–1.57)	0.05
5–6 months	105 (1.4)	368 (1.3)	1.25 (1.00–1.57)	0.05
*Antidepressants*				
Overall (prescribed within 6 months)	27 (0.4)	79 (0.3)	1.39 (0.88–2.18)	0.15
Index date to 1 month	4 (0.1)	24 (0.1)	0.66 (0.22–1.92)	0.44
1–2 months	14 (0.2)	33 (0.2)	1.62 (0.86–3.07)	0.14
2–3 months	14 (0.2)	38 (0.2)	1.34 (0.72–2.50)	0.36
3–4 months	14 (0.2)	47 (0.2)	1.08 (0.59–1.99)	0.80
4–5 months	12 (0.2)	46 (0.2)	1.01 (0.52–1.95)	0.98
5–6 months	14 (0.2)	46 (0.2)	1.30 (0.70–2.41)	0.40

Data are presented as *n* (%)

*ICU* intensive care unit; *CI* confidence interval

*Adjusted for age, sex, status of medical insurance, history of sleep disorders, history of anxiety disorders, history of mood disorders, history of post-traumatic stress disorders, and Charlson comorbidity index score

We also performed two additional sensitivity analyses; one excluded individuals whose spouses died within 6 months after the index date, and the other excluded spouses who withdrew their insurance within 6 months after the index date. The results were comparable with those of the main analyses (Additional file 1: Tables S4–S7, Figs. S1, S2).

## Discussion

Using a large non-elderly Japanese claims database, we investigated the proportions of ICU patients’ spouses who visited medical facilities for mental disorders related to PICS-F and the association between the ICU admission of patients and mental disorders in the ICU patients’ spouses. The proportion of mental disorders in the ICU patients’ spouses within 6 months after the patient’s ICU admission was 12.8%, which was slightly higher than that of the matched individuals.

One strength of our study is that it is the first to examine PICS-F using real-world data with a large number of ICU patients’ spouses. Another strength of our study is that we investigated the association between ICU admission of patients and mental disorders in the patients’ spouses by establishing a control group. Many studies have shown a high prevalence of mental disorders in such spouses; however, these studies could not show the actual association because they had no control group [7–16]. We also examined the proportions of individuals who received medications for mental disorders to avoid concerns regarding variations in diagnostic recording and coding among clinicians. The results of the secondary outcome were similar to those of the primary outcome, indicating the high robustness of our results.

The proportion of mental disorders in ICU patients’ spouses after the ICU admission was lower in the present study than in previous studies [7–15]. Moreover, although the proportions of mental disorders were significantly different between the ICU patients’ spouses and matched individuals, the difference was much smaller than expected. There are several possible reasons for this. First, differences in the study design may have caused a lower prevalence of spouses with mental disorders in our study. Our study was retrospective and used real-world data, whereas previous studies were prospective and the outcomes were measured with self-reported questionnaires or structured interviews. In the actual clinical setting, questionnaires or interviews are not routinely performed. This may explain why the proportion of spouses with mental disorders was lower in our study. Second, spouses may not consult primary care physicians or psychiatrists even if they suffer from mental disorders. In previous population-based studies of individuals with mental disorders, most individuals received no psychiatric treatment because of fear of stigmatization [27–29]. This may explain why the difference in the proportion of mental disorders between the two groups was small in our study. Third, although the spouses of the ICU patients were diagnosed with mental disorders by a questionnaire, many such spouses acquire resilience and recover from their mental disorders [30]. Indeed, the World Health Organization has stated that most bereaved individuals do not routinely require treatment for acute grief [31]. Thus, our results might indicate that some of the ICU patients’ spouses attained resilience and did not need treatment. Fourth, the prevalence of mental disorders in previous studies using questionnaires may be overestimated because of selection bias, non-response bias, and recall bias [7, 9, 11, 12]. Finally, the concept of PICS-F may not be widespread among the ICU practitioners, patients, and patients’ family members. Based on these possible reasons, we believe that it would be better to assess spouses’ mental state and, if indicated, ICU practitioners would be better to facilitate consultation between spouses and primary care physicians or psychiatrists in a timely manner.

### Potential limitations

Several limitations of this study should be acknowledged. First, most of the previous studies regarding PICS-F were based on questionnaires, whereas our study was based on ICD-10–based diagnoses. Diagnoses recorded in administrative claims databases are generally less accurate than those in planned prospective studies. Although diagnoses based on coding have high specificity and moderate sensitivity in Japan [32], diagnoses of mental disorders were not included in previous validation studies. However, as we previously discussed, we evaluated not only the diagnostic codes but also the prescribed medications to confirm the robustness of the ICD-10–based diagnoses [33]. Second, because our results represent the *proportion* of mental disorders in ICU patients’ spouses, the *incidence* of PICS-F may have been overestimated. This is because the proportion of mental disorders included (1) the spouses who had new mental disorders, (2) the spouses who had experienced mental disorders in the past and developed a relapse of mental disorders triggered by the ICU admission, and (3) spouses who had experienced mental disorders since the past. Additionally, we excluded spouses with a history of mental disorders; our results included only spouses who had new mental disorders. Therefore, the incidence of PICS-F may have been underestimated. Although our study revealed the association between ICU admission and an increasing incidence of spouses’ mental disorders, it may not represent the real-world incidence of PICS-F. Third, this study might have contained unmeasured confounders (e.g., socioeconomic status or psychological interventions) that were not recorded in the claims database. Fourth, the dropout rate was significantly higher in the exposure than non-exposure group. This difference may have led to underestimation of the negative effect of the patients’ ICU admissions on the patients’ spouses. However, we performed a sensitivity analysis that excluded spouses who withdrew their insurance within 6 months; the results of this analysis were almost identical to those of the original analysis. Finally, the generalizability of our findings is limited because the eligible patients in our study were only non-elderly patients. Previous studies have shown that young age of patients and their spouses is a risk factor for PICS-F [10]; thus, the effect size of the patients’ ICU admissions may vary depending on age groups.

## Conclusions

Spouses of patients who were admitted to ICUs were slightly more likely to have mental disorders than spouses of patients who were not admitted to ICUs. Further high-quality studies are needed to determine optimal and efficient strategies to identify individuals with PICS-F who actually need treatment and provide appropriate interventions.

## Supplementary Information

**Additional file 1: Table S1.** ICD-10 codes for Charlson comorbidity index score. **Table S2.** ICD-10 codes for main diagnoses necessitating admission in patients admitted to the ICU. **Table S3.** Baseline characteristics of ICU patients. **Table S4.** Proportions of ICU patients’ spouses and matched individuals who visited medical facilities for any mental disorders related to PICS-F after excluding those whose spouses died within 6 months from the index date. **Table S5.** Proportions of ICU patients’ spouses and matched individuals who visited medical facilities for any mental disorders related to PICS-F after excluding those who withdraw their insurance within 6 months from the index date. **Table S6.** Proportions of ICU patients’ spouses and matched individuals prescribed anxiolytics, hypnotics/sedatives, or antidepressants after excluding those whose spouses died within 6 months from the index date. **Table S7.** Proportions of ICU patients’ spouses and matched individuals prescribed anxiolytics, hypnotics/sedatives, or antidepressants after excluding those who withdraw their insurance within 6 months from the index date. **Figure S1.** Proportion of ICU patients’ spouses and matched individuals who prescribed psychotropic medications in 6 months before and after the index date, after excluding those whose spouses died within 6 months. Monthly percentages are based on a denominator of patients still registered in that month. **Figure S2.** Proportion of ICU patients’ spouses and matched individuals prescribed psychotropic medications in the 6 months before and after the index date, after excluding those who withdraw their insurance within 6 months from the index date. Monthly percentages are based on a denominator of patients still registered in that month.

## Data Availability

The data were used under license of JMDC Inc. for the current study; therefore, the data are not publicly available. For inquiries about access to the data set used in this study, please contact JMDC (https://www.jmdc.co.jp).

## Abbreviations

ATC: Anatomical Therapeutic Chemical Classification System
CI: Confidence interval
ICD-10: International Classification of Diseases, Tenth Revision
ICU: Intensive care unit
IQR: Interquartile range
OR: Odds ratio
PICS-F: Post-intensive care syndrome-family
SD: Standard deviation
