# Supplementary material for: Association between intensive care unit admission of a patient and mental disorders in the spouse: a retrospective matched-pair cohort study
Source: J Intensive Care. 2021 Oct 29;9:69. doi: 10.1186/s40560-021-00583-3 (PMC8555253; doi:10.1186/s40560-021-00583-3)

**Legends of Additional Material**

**e-Table 1.** ICD-10 codes for Charlson comorbidity index score

**e-Table 2.** ICD-10 codes for main diagnoses necessitating admission in patients admitted to the ICU

**e-Table 3.** Baseline characteristics of ICU patients

**e-Table 4.** Proportions of ICU patients’ spouses and matched individuals who visited medical facilities for any mental disorders related to PICS-F after excluding those whose spouses died within 6 months from the index date

**e-Table 5.** Proportions of ICU patients’ spouses and matched individuals who visited medical facilities for any mental disorders related to PICS-F after excluding those who withdraw their insurance within 6 months from the index date

**e-Table 6.** Proportions of ICU patients’ spouses and matched individuals prescribed anxiolytics, hypnotics/sedatives, or antidepressants after excluding those whose spouses died within 6 months from the index date

**e-Table 7.** Proportions of ICU patients’ spouses and matched individuals prescribed anxiolytics, hypnotics/sedatives, or antidepressants after excluding those who withdraw their insurance within 6 months from the index date

**e-Figure 1.** Proportion of ICU patients’ spouses and matched individuals who prescribed psychotropic medications in 6 months before and after the index date, after excluding those whose spouses died within 6 months. Monthly percentages are based on a denominator of patients still registered in that month.

**e-Figure 2.** Proportion of ICU patients’ spouses and matched individuals prescribed psychotropic medications in the 6 months before and after the index date, after excluding those who withdraw their insurance within 6 months from the index date. Monthly percentages are based on a denominator of patients still registered in that month.

**e-Table 1.** ICD-10 codes for Charlson comorbidity index score

| **Comorbidities** | **ICD-10 codes** |
| --- | --- |
| Myocardial infarction | I21, I22, I25.2 |
| Congestive heart failure | I09.9, I11.0, I13.0, I13.2, I25.5, I42.0, I42.5–I42.9, I43, I50, P29.0 |
| Peripheral vascular disease | I70, I71, I73.1, I73.8, I73.9, I77.1, I79.0, I79.2, K55.1, K55.8, K55.9, Z95.8, Z95.9 |
| Cerebrovascular disease | G45, G46, H34.0, I60–I69 |
| Dementia | F00–F03, F05.1, G30, G31.1 |
| Chronic pulmonary disease | I27.8, I27.9, J40–J47, J60–J67, J68.4, J70.1, J70.3 |
| Rheumatic disease | M05, M06, M31.5, M32–M34, M35.1, M35.3, M36.0 |
| Peptic ulcer disease | K25–K28 |
| Mild liver disease | B18, K70.0–K70.3, K70.9, K71.3–K71.5, K71.7, K73, K74, K76.0, K76.2–K76.4, K76.8, K76.9, Z94.4 |
| Diabetes without chronic complication | E10.0, E10.l, E10.6, E10.8, E10.9, E11.0, E11.1, E11.6, E11.8, E11.9, E12.0, E12.1, E12.6, E12.8, E12.9, E13.0, E13.1, E13.6, E13.8, E13.9, E14.0, E14.1, E14.6, E14.8, E14.9 |
| Diabetes with chronic complication | E10.2–E10.5, E10.7, E11.2–E11.5, E11.7, E12.2–E12.5, E12.7, E13.2–E13.5, E13.7, E14.2–E14.5, E14.7 |
| Hemiplegia or paraplegia | G04.1, G11.4, G80.1, G80.2, G81, G82, G83.0–G83.4, G83.9 |
| Renal disease | I12.0, I13.1, N03.2–N03.7, N05.2–N05.7, N18, N19, N25.0, Z49.0–Z49.2, Z94.0, Z99.2 |
| Any malignancy except malignant neoplasm of skin | C00–C26, C30–C34, C37–C41, C43, C45–C58, C60–C76, C81–C85 C88, C90–C97 |
| Moderate or severe liver disease | I85.0, I85.9, I86.4, I98.2, K70.4, K71.1, K72.1, K72.9, K76.5, K76.6, K76.7 |
| Metastatic solid tumor | C77–C80 |
| AIDS/HIV | B20–B22, B24 |

ICD-10, International Classification of Diseases, Tenth Revision; AIDS, acquired immunodeficiency syndrome; HIV, human immunodeficiency virus

The comorbidities were identified if relevant ICD-10 diagnostic codes appeared at least once during the 6 months before the index date.

**e-Table 2.** ICD-10 codes for main diagnoses necessitating admission in patients admitted to the ICU

| **Diagnosis** | **ICD-10 codes** |
| --- | --- |
| Sepsis | A02.0, A04, A05, A08, A09, A15–A18, A19, A27, A28, A31.0, A31.1, A31.8, A31.9, A32, A35, A37, A39–A43, A46, A48.0–A48.3, A48.8, A49, A52–A54, A69, B35, B36, B37.1, B37.2, B37.4, B37.5–B37.9, B44.0–B44.2, B44.7–B46, B48, B49, G00–G09, G45, I20, I21, I23, I249, I30, I33, I46, I49.0, I60, I61, I62.1, I62.9, I63, I64, I80, J01–J06, J13–J18, J20–J22, J44.0, J44.1, J47, J85, J86, K35–K37, K57, K61, K63.0, K63.1, K65, K67, K75.0, K75.1, K80.0, K80.3, K80.4, K80.8, K81.0, K83.0, K91.8, L00, L03, L04, L08, L72.6, L88, M00, M86, N10–N12, N15.1, N15.9, N16.0, N30, N34, N39.0, N41, N70–N73, N76, N77, R95, R96, R98, T81.4, T82.6, T82.7, T83.5, T84.5–T84.7, T85.7 |
| Stroke | I60, I61, I621, I629, I63, I64, G45 |
| Acute coronary syndrome | I20, I21, I23, I24.9 |
| Congestive heart failure | I09.9, I11.0, I13.0, I13.2, I25.5, I42.0, I42.5–I43, I50, P29.0 |
| Trauma | S00–T14 |
| Aortic diseases | I71 |

ICD-10, International Classification of Diseases Tenth Revision; ICU, intensive care unit

The main diagnoses were identified if relevant ICD-10 diagnostic codes appeared in the main diagnoses during hospitalization requiring ICU admission.

**e-Table 3.** Baseline characteristics of ICU patients

| **Characteristics** | **ICU patients (n = 8,490)** |
| --- | --- |
| Age, years, mean (SD) | 54.9 (9.9) |
| Female | 2,996 (35.3) |
| Medical insurance |  |
| Independents | 3,108 (36.6) |
| Dependents | 5,382 (63.4) |
| History of any mental disorders | 1,849 (21.8) |
| History of mood disorders | 769 (9.1) |
| History of anxiety disorders | 539 (6.3) |
| Charlson comorbidity index |  |
| 0 | 687 (8.1) |
| 1 | 1,570 (18.5) |
| 2 | 1,460 (17.2) |
| 3 | 1,305 (15.4) |
| ≥4 | 3,468 (40.8) |
| **Main diagnosis necessitating admission** |  |
| Sepsis | 4,520 (53.2) |
| Stroke | 1,953 (23.0) |
| Acute coronary syndrome | 1,510 (17.8) |
| Trauma | 800 (9.4) |
| Aortic diseases | 479 (5.6) |
| Congestive heart failure | 422 (5.0) |
| **Medical treatment and intervention** |  |
| Mechanical ventilation | 2,614 (30.8) |
| Vasopressors | 3,861 (45.5) |
| Renal replacement therapy | 429 (5.1) |
| Antibiotics | 5,542 (65.3) |
| General anesthesia | 3,471 (40.9) |
| Percutaneous coronary intervention | 970 (11.4) |
| Intra-aortic balloon pumping | 307 (3.6) |
| Percutaneous cardiopulmonary support | 114 (1.3) |
| Defibrillation | 244 (2.9) |
| Chest compression | 250 (2.9) |
| **Outcomes** |  |
| Death within 6 months after the index date | 586 (6.9) |
| Length of stay, days, median (IQR) | 16 (10–28) |
| Length of ICU stay, days, median (IQR) | 3 (2–7) |

Unless otherwise stated, data are presented as number (%).

ICU, intensive care unit; IQR, interquartile range; SD, standard deviation

**e-Table 4.** Proportions of ICU patients’ spouses and matched individuals who visited medical facilities for any mental disorders related to PICS-F after excluding those whose spouses died within 6 months from the index date

| **Period of time** | **ICU patients’ spouses (n = 8,243)** | **Matched individuals**  **(n = 33,707)** | **Adjusted odds ratio (95% CI)*** | **P-value** |
| --- | --- | --- | --- | --- |
| Overall (index date to 6 months) | 1,059 (12.8) | 3,790 (11.2) | 1.29 (1.03–1.42) | 0.02 |
| Index date to 1 month | 683 (8.3) | 2,440 (7.2) | 1.16 (0.93–1.46) | 0.17 |
| 1 to 2 months | 637 (7.7) | 2,419 (7.2) | 1.24 (1.02–1.51) | 0.03 |
| 2 to 3 months | 597 (7.2) | 2,327 (6.9) | 1.02 (0.85–1.23) | 0.79 |
| 3 to 4 months | 608 (7.4) | 2,315 (6.9) | 1.10 (0.92–1.32) | 0.30 |
| 4 to 5 months | 577 (7.0) | 2,209 (6.6) | 1.00 (0.84–1.20) | 1.00 |
| 5 to 6 months | 562 (6.8) | 2,190 (6.5) | 0.99 (0.83–1.18) | 0.93 |

Data are presented as n (%).

*Adjusted for age, sex, status of medical insurance, history of sleep disorders, history of anxiety disorders, history of mood disorders, history of post-traumatic stress disorders, and Charlson comorbidity index score

PICS-F, post-intensive care syndrome-family; ICU, intensive care unit; CI, confidence interval

**e-Table 5.** Proportions of ICU patients’ spouses and matched individuals who visited medical facilities for any mental disorders related to PICS-F after excluding those who withdraw their insurance within 6 months from the index date

| **Period of time** | **ICU patients’ spouses (n = 7,248)** | **Matched individuals**  **(n = 29,423)** | **Adjusted odds ratio (95% CI)*** | **P-value** |
| --- | --- | --- | --- | --- |
| Overall (index date to 6 months) | 162 (2.2) | 471 (1.6) | 1.41 (1.17–1.70) | <0.001 |
| Index date to 1 month | 24 (0.3) | 50 (0.2) | 2.07 (1.23–3.47) | 0.006 |
| 1 to 2 months | 56 (0.8) | 102 (0.3) | 2.20 (1.57–3.10) | <0.001 |
| 2 to 3 months | 55 (0.8) | 142 (0.5) | 1.56 (1.12–2.15) | 0.007 |
| 3 to 4 months | 75 (1.0) | 178 (0.6) | 1.75 (1.32–2.32) | <0.001 |
| 4 to 5 months | 69 (1.0) | 182 (0.6) | 1.61 (1.20–2.14) | 0.001 |
| 5 to 6 months | 68 (0.9) | 211 (0.7) | 1.34 (1.01–1.80) | 0.04 |

Data are presented as n (%).

*Adjusted for age, sex, status of medical insurance, history of sleep disorders, history of anxiety disorders, history of mood disorders, history of post-traumatic stress disorders, and Charlson comorbidity index score

ICU, intensive care unit; CI, confidence interval

**e-Table 6.** Proportions of ICU patients’ spouses and matched individuals who prescribed anxiolytics, hypnotics/sedatives, or antidepressants after excluding those whose spouses died within 6 months from the index date

| **Period of time** | **ICU patients’ spouses (n = 8,243)** | **Matched individuals**  **(n = 33,707)** | **Adjusted odds ratio (95% CI) *** | **P-value** |
| --- | --- | --- | --- | --- |
| **Anxiolytics and hypnotics/sedatives** |  |  |  |  |
| Overall (prescribed within 6 months) | 1,043 (12.7) | 3,595 (10.7) | 1.19 (1.07–1.32) | 0.001 |
| Index date to 1 month | 690 (8.4) | 2,272 (6.7) | 1.33 (1.13–1.55) | <0.001 |
| 1 to 2 months | 661 (8.0) | 2,211 (6.6) | 1.32 (1.14–1.53) | <0.001 |
| 2 to 3 months | 593 (7.2) | 2,207 (6.5) | 1.09 (0.94–1.26) | 0.25 |
| 3 to 4 months | 572 (6.9) | 2,122 (6.3) | 1.06 (0.91–1.22) | 0.45 |
| 4 to 5 months | 562 (6.8) | 2,031 (6.0) | 1.16 (1.00–1.34) | 0.05 |
| 5 to 6 months | 545 (6.6) | 2,022 (6.0) | 1.10 (0.95–1.28) | 0.20 |
| **Antidepressants** |  |  |  |  |
| Overall (prescribed within 6 months) | 223 (2.7) | 832 (2.5) | 1.17 (0.86–1.59) | 0.31 |
| Index date to 1 month | 180 (2.2) | 695 (2.1) | 1.03 (0.65–1.65) | 0.89 |
| 1 to 2 months | 175 (2.1) | 676 (2.0) | 1.30 (0.86–1.95) | 0.21 |
| 2 to 3 months | 165 (2.0) | 661 (2.0) | 1.19 (0.81–1.76) | 0.38 |
| 3 to 4 months | 153 (1.8) | 646 (1.9) | 0.96 (0.65–1.41) | 0.83 |
| 4 to 5 months | 148 (1.7) | 616 (1.8) | 1.01 (0.68–1.50) | 0.96 |
| 5 to 6 months | 143 (1.7) | 604 (1.8) | 1.03 (0.69–1.52) | 0.90 |

Data are presented as n (%).

*Adjusted for age, sex, status of medical insurance, history of sleep disorders, history of anxiety disorders, history of mood disorders, history of post-traumatic stress disorders, and Charlson comorbidity index score

ICU, intensive care unit; CI, confidence interval

**e-Table 7.** Proportions of ICU patients’ spouses and matched individuals who prescribed anxiolytics, hypnotics/sedatives, or antidepressants after excluding those who withdraw their insurance within 6 months from the index date

| **Period of time** | **ICU patients’ spouses (n = 6,716)** | **Matched individuals**  **(n = 28,763)** | **Adjusted odds ratio (95% CI) *** | **P-value** |
| --- | --- | --- | --- | --- |
| **Anxiolytics and hypnotics/sedatives** |  |  |  |  |
| Overall (prescribed within 6 months) | 876 (13.0) | 3,125 (10.9) | 1.24 (1.11–1.39) | <0.001 |
| Index date to 1 month | 552 (8.2) | 1,903 (6.6) | 1.41 (1.18–1.68) | <0.001 |
| 1 to 2 months | 544 (8.1) | 1,902 (6.6) | 1.41 (1.20–1.66) | <0.001 |
| 2 to 3 months | 517 (7.7) | 1,966 (6.8) | 1.18 (1.01–1.38) | 0.04 |
| 3 to 4 months | 515 (7.7) | 1,953 (6.8) | 1.08 (0.92–1.27) | 0.33 |
| 4 to 5 months | 530 (7.9) | 1,922 (6.7) | 1.26 (1.07–1.48) | 0.004 |
| 5 to 6 months | 528 (7.9) | 1,957 (6.8) | 1.19 (1.02–1.39) | 0.03 |
| **Antidepressants** |  |  |  |  |
| Overall (prescribed within 6 months) | 183 (2.7) | 703 (2.4) | 1.15 (0.85–1.56) | 0.37 |
| Index date to 1 month | 146 (2.2) | 580 (2.0) | 1.23 (0.73–2.08) | 0.44 |
| 1 to 2 months | 146 (2.2) | 582 (2.0) | 1.22 (0.82–1.83) | 0.31 |
| 2 to 3 months | 144 (2.1) | 587 (2.0) | 1.14 (0.78–1.68) | 0.50 |
| 3 to 4 months | 142 (2.1) | 591 (2.1) | 0.90 (0.61–1.33) | 0.60 |
| 4 to 5 months | 141 (2.1) | 586 (2.0) | 0.95 (0.64–1.40) | 0.79 |
| 5 to 6 months | 142 (2.1) | 587 (2.0) | 0.97 (0.65–1.43) | 0.87 |

Data are presented as n (%).

*Adjusted for age, sex, status of medical insurance, history of sleep disorders, history of anxiety disorders, history of mood disorders, history of post-traumatic stress disorders, and Charlson comorbidity index score

ICU, intensive care unit; CI, confidence interval

**e-Figure 1.** Proportion of ICU patients’ spouses and matched individuals who prescribed psychotropic medications in 6 months before and after the index date, after excluding those whose spouses died within 6 months. Monthly percentages are based on a denominator of patients still registered in that month.


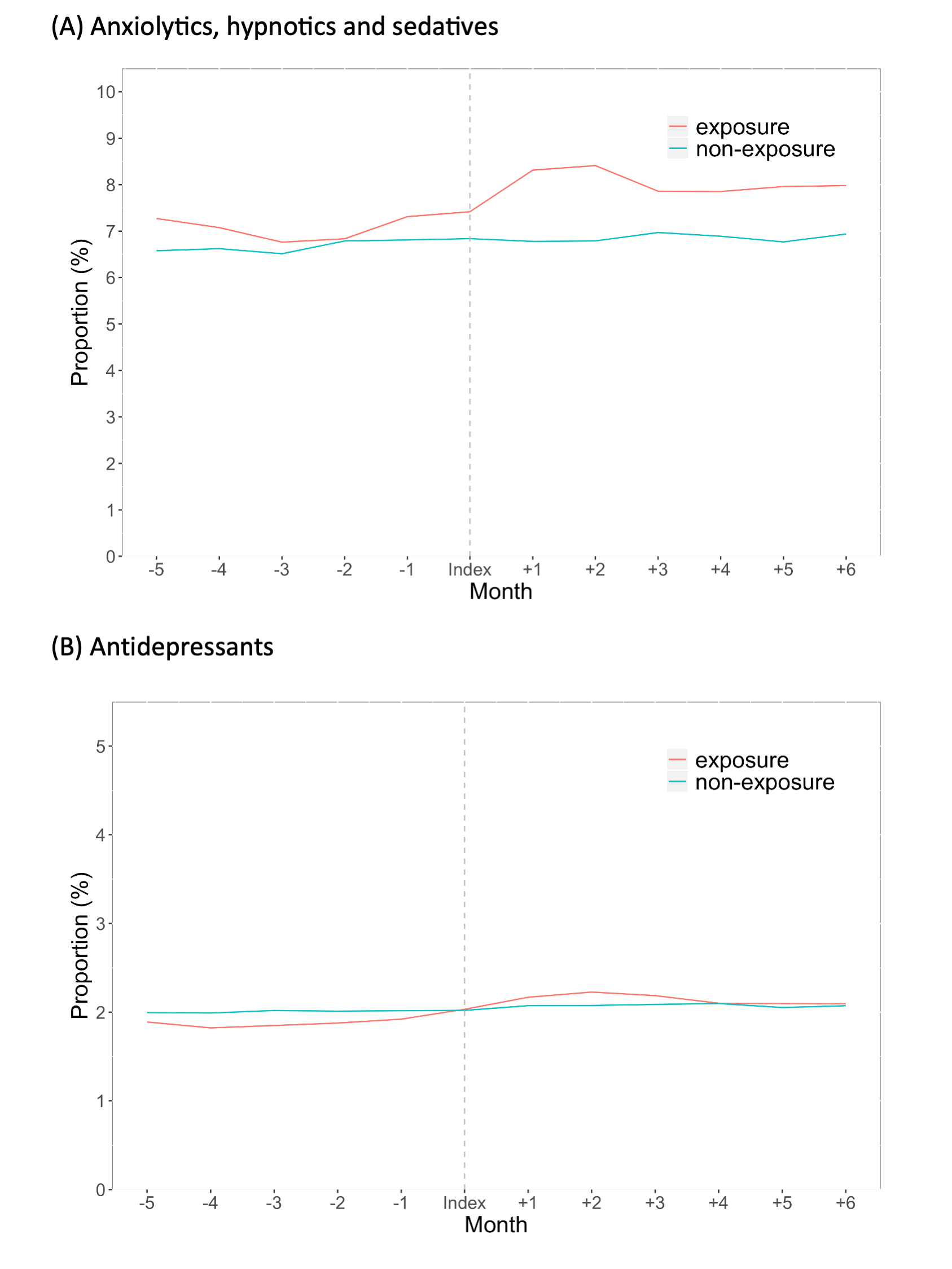
**e-Figure 2.** Proportion of individuals prescribed psychotropic medications in the 6 months before and after the index date, after excluding those who withdraw their insurance within 6 months from the index date. Monthly percentages are based on a denominator of patients still registered in that month.


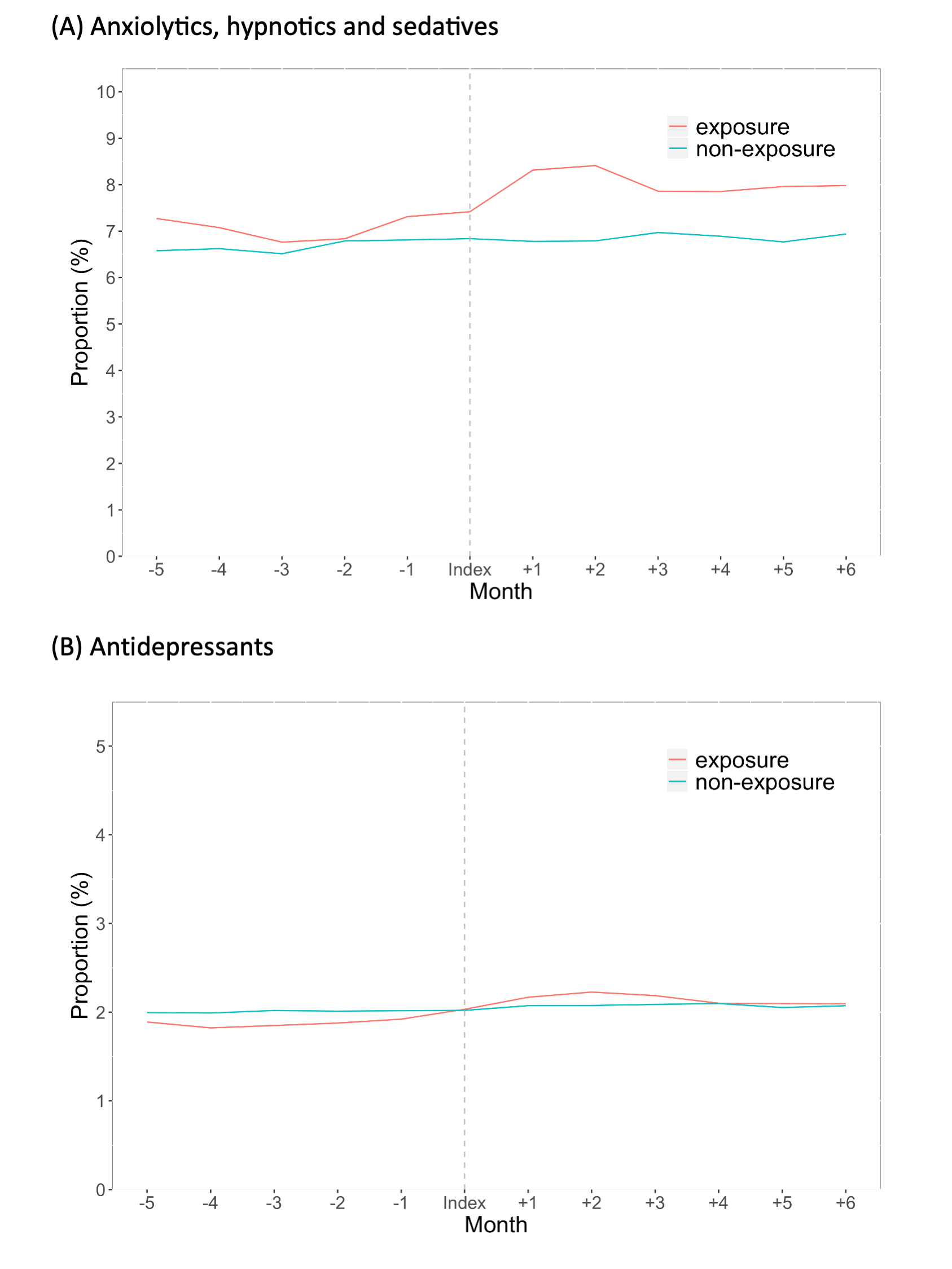

Supplement: Supplementary file 1 — Additional file 1: Table S1. ICD-10 codes for Charlson comorbidity index score. Table S2. ICD-10 codes for main diagnoses necessitating admission in patients admitted to the ICU. Table S3. Baseline characteristics of ICU patients. Table S4. Proportions of ICU patients’ spouses and matched individuals who visited medical facilities for any mental disorders related to PICS-F after excluding those whose spouses died within 6 months from the index date. Table S5. Proportions of ICU patients’ spouses and matched individuals who visited medical facilities for any mental disorders related to PICS-F after excluding those who withdraw their insurance within 6 months from the index date. Table S6. Proportions of ICU patients’ spouses and matched individuals prescribed anxiolytics, hypnotics/sedatives, or antidepressants after excluding those whose spouses died within 6 months from the index date. Table S7. Proportions of ICU patients’ spouses and matched individuals prescribed anxiolytics, hypnotics/sedatives, or antidepressants after excluding those who withdraw their insurance within 6 months from the index date. Figure S1. Proportion of ICU patients’ spouses and matched individuals who prescribed psychotropic medications in 6 months before and after the index date, after excluding those whose spouses died within 6 months. Monthly percentages are based on a denominator of patients still registered in that month. Figure S2. Proportion of ICU patients’ spouses and matched individuals prescribed psychotropic medications in the 6 months before and after the index date, after excluding those who withdraw their insurance within 6 months from the index date. Monthly percentages are based on a denominator of patients still registered in that month. [file 40560_2021_583_MOESM1_ESM.docx]
